# Methanogenic Community Dynamics during Anaerobic Utilization of Agricultural Wastes 

**Published:** 2012

**Authors:** A. M. Ziganshin, E. E. Ziganshina, S. Kleinsteuber, J. Pröter, O. N. Ilinskaya

**Affiliations:** Kazan (Volga Region) Federal University, Kremlyovskaya Str., 18, Kazan, Russia, 420008; UFZ-Helmholtz Centre for Environmental Research, Permoser Str., 15, Leipzig, Germany 04318; German Biomass Research Centre, Torgauer Str., 116, Leipzig, Germany, 04347

## Abstract

This work is devoted to the investigation of the methanogenic archaea involved in anaerobic digestion of cattle manure and maize straw on the basis of terminal restriction fragment length polymorphism (T-RFLP) analysis of archaeal 16S rRNA genes. The biological diversity and dynamics of methanogenic communities leading to anaerobic degradation of agricultural organic wastes with biogas production were evaluated in laboratory-scale digesters. T-RFLP analysis, along with the establishment of archaeal 16S rRNA gene clone libraries, showed that the methanogenic consortium consisted mainly of members of the genera*Methanosarcina*and*Methanoculleus,*with a predominance of*Methanosarcina*spp. throughout the experiment.

## INTRODUCTION 

One of the most effective methods for reducing the negative effects of the waste from the agricultural and processing industries on the environment is their anaerobic digestion. Anaerobic digestion of wastes is accompanied by the destruction of most organic components and production of biogas consisting of methane (50–75%) and carbon dioxide (25–50%), with trace amounts of other components. In contrast to bioethanol and biodiesel mostly produced from energy crops, biogas is obtained during utilization of residual biomass and various organic wastes [1–7], such as cattle manure. However, due to the low biodegradability of manure, its utilization in anaerobic reactors is characterized by an insignificant biogas yield. Anaerobic co-digestion of manure and plant biomass promotes substrate hydrolysis, optimizes the distribution of nutrients in the bioreactor, thus activating microbial growth and the biomethane yield [8, 9]. The co-digestion of several different substrates has been actively investigated over the past years [9–13]. 

The first three stages of anaerobic co-digestion (hydrolysis, acidogenesis, and acetogenesis) are performed by bacterial communities; the fourth stage is performed by aceticlastic and hydrogenotrophic methanogens, which consume acetate, molecular hydrogen, and carbon dioxide to produce methane [1, 6, 14]. 

Independently of the mode of digestion (psychro-, meso-, or thermophilic) and feedstock composition, the major participants in methanogenesis are the members of the orders of *Methanomicrobiales * and/or *Methanosarcinales* [2, 5, 7, 15–18]. However, there is a lack of information about the changes in microbial association during methanogenic fermentation. 

The present study was devoted to the investigation of pathways for utilization of agricultural wastes (manure and maize straw) with biogas production in laboratory-scale biogas reactors and to studying the diversity, structure, and dynamics of the methanogenic communities involved in this process using modern methods of molecular biology. The determination of the composition and dynamics of the microbial communities in biogas reactors, jointly with the analysis of substrate destruction, is aimed at revealing the potential for intensification of the anaerobic process. The use of the universal phylogenic marker 16S rRNA and T-RFLP (terminal restriction fragment length polymorphism) will contribute to the study of the composition and temporal changes to the microbial consortium. 

## MATERIALS AND METHODS 

**Digester configurations **

Table 1 lists the main technological parameters of the anaerobic processing of cattle manure and maize straw. All bioreactors were run under mesophilic conditions (38°C). The bioreactors R 4.13 and R 4.14 were loaded with cattle manure and maize straw; the bioreactors R 4.15 and R 4.16, with cattle manure and extruded maize straw. Feeding a new portion of substrate and unloading of the digested mixture were performed daily; the volume of the digesting mixture was maintained at the level of 30 L; the hydraulic retention time (HRT) was kept constant in the bioreactors (35 days). The biogas yield, composition and pH were analyzed daily, whereas the concentrations of organic acids and ammonium ions were measured twice a week. 

**Analytical methods **

Biogas production was monitored using Ritter TG 05 drum-type gas meters (Bochum, Germany); biogas composition was measured by an infrared landfill gas analyzer, GA 94 (Ansyco, Germany). Ammonium concentration was analyzed by coloring of the liquid phase of the bioreactor contents with Nessler’s reagent on a spectrophotometer DR/2000 (HachCompany, USA) at 425 nm. 

The total acid capacity was determined by titration with 0.025–0.1 M H _2_ SO _4_ in a pH range from 4.5 to 3.5 using a Titration Excellence T90 titrator (Mettler-Toledo, Switzerland). The concentration of volatile fatty acids (VFA) was analyzed by gas chromatography using a 5890 series II GC (Hewlett Packard, USA) equipped with an HS40 automatic headspace sampler (Perkin Elmer, USA) and an Agilent HP-FFAP column (30 m×0.32 mm×0.25 μm), as described previously [7]. 

**DNA extraction and purification **

Samples were collected from four reactors once a month and were immediately used for DNA extraction and purification. The digested biomass mixture was centrifuged at 20,000 g for 10 min. Total DNA was subsequently extracted and purified using a FastDNA Spin Kit for soil (Qbiogene, Germany) according to the manufacturer’s recommendations. The total amount of extracted and purified DNA was measured on a NanoDrop ND-1000 UV–visible spectrophotometer (PeqLab, Germany). 

**Amplification, cloning and sequencing of archaeal 16S rRNA **

All molecular manipulations were performed according to our previous work [7]. Archaeal 16S rRNA genes were amplified from the total DNA as a template in a DNA Engine Tetrad 2 Peltier Thermal Cycler (Bio-Rad) using a combination of universal primers UniArc21F (5’-TTCYGKTTGATCCYGSCRG-3’) and UniArc931R (5’-CCCGCCAATTCCTTTHAG-3’) and 2 × *Taq* MasterMix (Qiagen, Germany). The composition of the reaction mixture was as follows: 6 µL of 2 × *Taq* MasterMix, 0.5 µL of UniArc21F (5 pmol/µL), 0.5 µL of UniArc931R (5 pmol/µL), 4 µL of H _2_ O, and 1 µL of 100-fold diluted DNA template (equivalent of 1–3 ng). The amplification was started with denaturation at 95°C for 5 min, followed by 35 cycles: denaturation at 94° C for 1 min, annealing at 54° C for 1 min, and elongation at 72° C for 2 min. The final elongation was carried out at 72° C for 2 min. 

The PCR products were purified using a QIAGEN PCR Cloning Kit (QIAGEN, Germany). The presence of inserts of archaeal 16S rRNA genes of the desired size in positive clones after cloning was analyzed using the vector-specific primers M13uni(–21) (5’-TGTAAAACGACGGCCAGT-3’) and M13rev(–29) (5’-CAGGAAACAGCTATGACC-3’). 1 µL of the M13-amplicons were further treated with HaeIII endonuclease (New England Biolabs, Germany) and separated by Phor-agarose gel electrophoresis (Biozym, Germany). The lengths of restricted fragments were analyzed using the Phoretix ^TM^ 1D Database Version 2.00 and Phoretix ^TM^ 1D Advanced Version 5.20 (Nonlinear Dynamics, Great Britain) software; clones were grouped into clusters, and dendrograms were constructed. The representative clones from large clusters were selected to further determine their nucleotide sequences. 

The PCR products of the representative clones were purified using a Promega PCR Purification Kit (Promega, USA). The nucleotide sequences of the 16S rRNA genes were determined using a BigDye ^TM ^ Terminator Cycle Sequencing Ready Reaction Kit 1.1 on an ABIPRISM 3100 Genetic Analyzer automated sequencer (Applied Biosystems). The POP-6 ^TM^ polymer was used as a separation matrix. The BLAST tool (http://blast.ncbi.nlm.nih.gov/Blast.cgi) [21] was employed to search for similar sequences in the GenBank database. The Ribosomal Database Project (http://rdp.cme.msu.edu) [22] was used for taxonomic assignment. 

**T-RFLP analysis **

The T-RFLP analysis was performed in accordance with our previous work [7]. The archaeal 16S rRNA genes were amplified using a universal primer pair UniArc21F-FAM and UniArc931R and 2 × *Taq* MasterMix (Qiagen, Germany) with the PCR parameters as described above. The forward primer UniArc21F-FAM was marked with a FAM fluorophor (phosphoramidite fluorochrome-5-carboxyfluorescein) at the 5’ end. The amplicons of the archaeal 16S rRNA genes containing FAM fluorophor were purified using a SureCleanPlus kit (Bioline, Germany) and treated with the MseI and HaeIII restrictases (New England Biolabs, Germany). After a 16-hour-long incubation at 37 ^o^ C, DNA fragments were precipitated with 3 M sodium acetate (pH 5.5) and absolute ethanol. The supernatant was removed; the precipitate was dried in vacuum, and the resulting DNA fragments were resuspended in 10 μL of Hi-Di formamide containing 0.25 µL of GeneScan-500 ROX™ STANDARD or MapMarker® 1000 size standard. The samples were denatured at 95°C for 5 min, cooled on ice (approximately for 5 min), and analyzed on an ABIPRISM 3100 Genetic Analyzer (Applied Biosystems). The POP-6 ^TM^ polymer was used as a separation matrix. The resulting T-RLFP patterns were analyzed using the GeneMapper V3.7 software (Applied Biosystems). The theoretical T-RF values of the representative phylotypes listed in the clone library were calculated using the NEB cutter Version 2.0 (http://tools.neb.com/NEBcutter2) and confirmed experimentally by T-RFLP analysis using the corresponding clones as templates. 

## RESULTS AND DISCUSSION 

**Table 1:** Main configurations of anaerobic digestion of cattle manure and maize straw

Diges­ter*	Organic loading rate**,g_oTS_ day^–1^	Substrate composition, g day^–1^			Biogas yield under standard conditions, L g^–1^_oTS_	Biogas composition			pH	Acid capacity, g L^–1^	NH_4_^+^-N, g L^–1^
		cattle manure	straw	total***		CH_4_, %	CO_2,_%	H_2_S, ppm.			
R 4.13	74.1	723.6	28.2	857	0.40	58.7	40.2	3450	7.63	1.49	1.20
	71.2	518.7	26.3	857	0.36	59.8	38.7	2216	7.50	1.90	1.24
	71.7	694.6	26.3	857	0.33	55.6	42.9	2145	7.61	1.80	1.16
R 4.14	74.1	723.6	28.2	857	0.40	59.3	39.8	4183	7.66	1.42	1.22
	71.2	518.7	26.3	857	0.38	58.4	40.2	1928	7.53	1.66	1.28
	71.7	694.6	26.3	857	0.37	56.7	42.1	2092	7.58	1.43	1.31
R 4.15	72.1	723.6	83.7	857	0.39	58.1	41.1	~5000	7.75	1.54	1.47
	68.6	518.7	78.1	857	0.39	59.3	39.2	2234	7.56	1.28	1.39
	69.1	694.6	78.1	857	0.39	56.8	42.6	2373	7.74	1.37	1.26
R 4.16	72.1	723.6	83.7	857	0.41	58.6	40.6	4558	7.76	1.51	1.54
	68.6	518.7	78.1	857	0.38	59.0	40.1	2056	7.54	1.53	1.36
	69.1	694.6	78.1	857	0.39	57.2	41.5	3155	7.61	1.37	1.27

* Digester parameters are presented at three sampling times, when methanogenic communities were analyzed (except for biogas yield, biogas composition, and pH, with values averaged over 1 week).

** Organic total solids.

*** Water was added to final concentration of 857 ml day ^–1^ .

The use of renewable energy sources, in particular various types of organic waste, is an essential aspect of “green technologies” for biofuel production [1]. The aim of this work was to investigate the dynamics of methanogenic associations during the conversion of cattle manure and maize straw in model mesophilic digesters. 

Table 1 lists the major operational parameters of the anaerobic digestion of agricultural waste as substrates. Anaerobic biomass destruction was carried out in four laboratory-scale digesters with an operating volume of 30 L at 38 ^о^ C. In the reactors R 4.13 and R 4.14, cattle manure and maize straw were co-digested; the reactors R 4.15 and R 4.16 were loaded with manure and extruded maize straw. The organic loading rate (OLR) was varied from 71.2 to 74.1 g _oTS_ day ^-1^ (organic total solids) in the reactors R 4.13 and R 4.14. In the reactors R 4.15 and R 4.16, the OLR was lower and varied from 68.6 to 72.1 g _oTS_ day ^-1^ . Throughout the experiment the HRT was kept constant (35 days). Depending on the particular feedstock, the biogas yield varied from 0.33 to 0.41 L g ^-1 ^_oTS_ with a methane content of 56–60%. As can be seen in *Table 1,* pH in all bioreactors was maintained at approximately 7.5–7.8; acid capacity ranged between 1.3 and 1.9 g L ^-1^ , and ammonium concentration varied from 1.2 to 1.5 g L ^-1^ . These parameters are favorable for methanogenesis [23]. 

**Table 2:** Results of sequencing of archaeal 16S rRNA gene clones and experimentally determined terminal restriction fragments (T-RF)

Clone, bp	Nearest relative (GenBank accession No) / coincidence %	Taxonomic status in accordance with RDP 10	MseI-T-RF, bp	HaeIII-T-RF, bp
ar_B9 (863)	Uncultured archaeon clone: FA69 (AB494258) / 99%	*Methanoculleus *sp.	37	67
ar_A1 (864)	Uncultured*Methanoculleus*sp. Clone: DMMR219 (HM218939) / 99%	*Methanoculleus *sp.	36	67
OTU 1		*Methanoculleus sp. I*	36/37	67
ar_A2 (863)	Uncultured archaeon clone: MTSArc_G8 (EU591664) / 99%	*Methanoculleus *sp.	499	67
OTU 2		*Methanoculleus sp. II*	499	67
ar_E12 (864)	Uncultured archaeon clone: WA50 (AB494245) / 100%	*Methanocorpusculum *sp.	97	241
OTU 3		*Methanocorpusculum sp. *	97	241
ar_E10 (567)	Uncultured euryarchaeote clone: B35_F_A_A05 (EF552199) / 99%	*Methanosarcina *sp.	557	220
ar_H2 (873)	Uncultured euryarchaeote clone: B35_F_A_A05 (EF552199) / 99%	*Methanosarcina *sp.	557	220
OTU 4		*Methanosarcina sp. I*	557	220
ar_E6 (873)	Uncultured archaeon clone: SA42 (AB494252) / 99%	*Methanosarcina *sp.	859	220
ar_F10 (873)	Uncultured archaeon clone: SA42 (AB494252) / 99%	*Methanosarcina *sp.	858	220
OTU 5		*Methanosarcina sp. II*	858/859	220
ar_G8 (874)	Uncultured archaeon clone: SA42 (AB494252) / 99%	*Methanosarcina *sp.	877	220
OTU 6		*Methanosarcina sp.III*	877	220

The biological diversity and dynamics of methanogenic communities digesting cattle manure and maize straw were investigated by amplification, cloning, restriction analysis, and sequencing of the archaeal 16S rRNA genes. The methanogenic association structure was determined at three sampling points with a 1-month interval. 

Amplification, cloning, sequencing of archaeal 16S rRNA, and T-RLFP analysis revealed a relatively large diversity of archaeal species in the reactors. During the T-RLFP analysis of archaeal 16S rRNA gene amplicons containing FAM flurophor were treated with endonucleases MseI and HaeIII. Belonging of the peaks in T-RLFP patterns to certain phylogenic groups was determined by the length of the terminal restriction fragments (T-RF) of 16S rRNA gene clones. In total, 9 clones were selected from the clone library for sequencing. The clones were classified into 6 operational taxonomic units (OTUs) on the basis of their T-RF lengths ( *Table* 2). Three phylotypes were attributed to the order *Methanomicrobiales* (OTU 1, OTU 2, OTU 3), and three were attributed to the order *Methanosarcinales* (OTU 4, OTU 5, OTU 6). Up to 22 different T-RFLP profiles (with abundance of more than 1%) were detected by T-RLFP analysis of 16S rRNA genes using MseI restrictase. Since the main T-RFs in the reactors were identified, we identified the methanogens playing the key role in biogas production. 

**Fig. 1:**
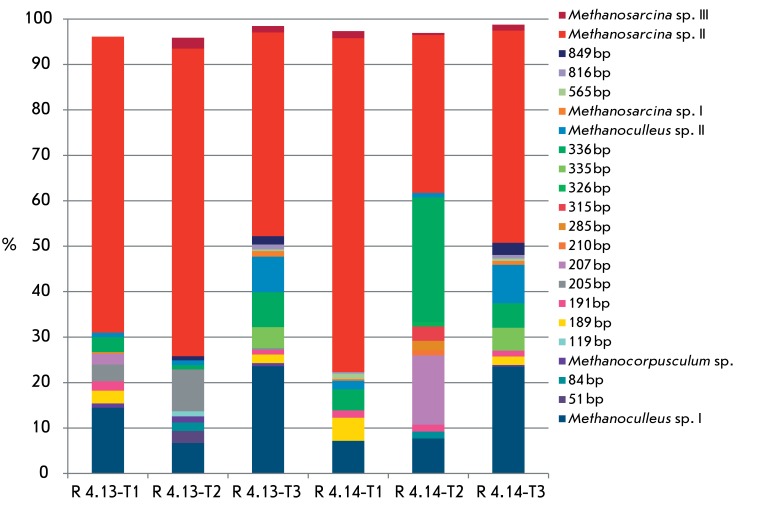
Dynamics of methanogenic communities in the digesters R 4.13 and R 4.14 based on T-RFLP analysis (determined with the restriction enzyme MseI)

*Fig. 1* shows the distribution of groups of methanogens (community dynamics) during anaerobic digestion of manure and straw (R 4.13 and R 4.14). This distribution was obtained based on MseI restriction profiles (results of HaeIII restriction are not shown). In the first sample, when the organic loading rate was 74.1 g _oTS_ day ^-1^ , the T-RFLP analysis revealed the predominance of methanogens of the genus *Methanosarcina* and hydrogenotrophic methanogens of the genus *Methanoculleus* in the archaeal community of the bioreactors R 4.13 and R 4.14. Thus, the total ratio of representatives of *Methanosarcina* sp. (OTU 4, OTU 5, and OTU 6) and *Methanoculleus * sp. (ОТU 1, ОТU 2) was 65 and 15%, respectively, of the total T-RF peak areas in the reactor R 4.13. In the reactor R 4.14, methanogens of the genera * Methanosarcina* (75%) and *Methanoculleus * (9%)were detected. Other archaeal members with low abundance (1–3%) were classified into the minor groups. A decrease in OLR to 71.2 g _oTS_ day ^–1^ with a subsequent increase to 71.7 g _oTS_ day ^–1^ resulted in a change in the composition of the microbial community. Thus, the relative abundance of members of the genus *Methanosarcina* (OTU 4, OTU 5, OTU 6) in the two next sampling points reached 70/47% and 35/49% values for the reactors R 4.13 and R 4.14, respectively. The relative abundance of the species of the genus *Methanoculleus* (ОТU 1, ОТU 2) in the reactors R 4.13 and R 4.14 was 8/31 and 9/32%, respectively (two next sampling points). 

Hydrogentrophic methanogens from the genus *Methanocorpusculum* were found among the minor associations and they comprised less than 2% of the total T-RF area. Furthermore, the major peak corresponding to 336 bp was detected in T-RLFP patterns; however, this phylotype was not present among the cloned archaeal 16S rRNA genes, and, hence, it was assigned to the unidentified group of the methanogenic community. 

**Fig. 2:**
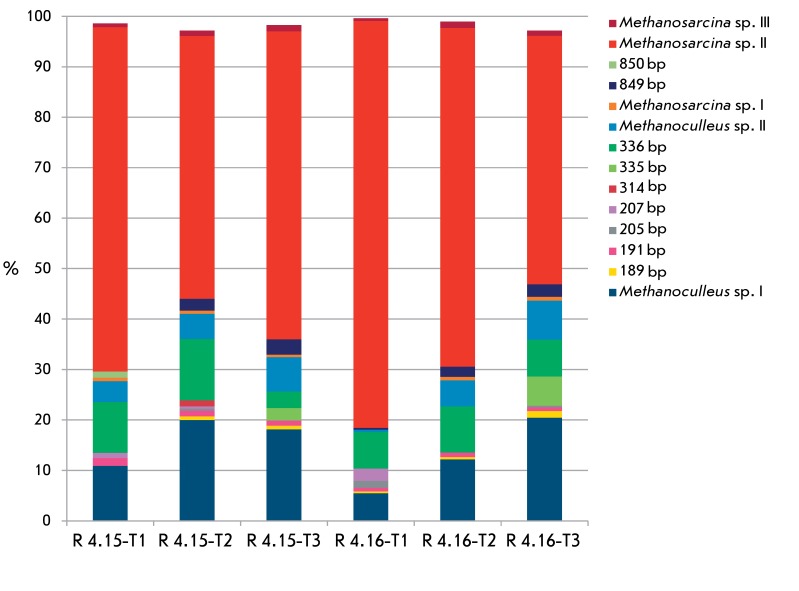
Dynamics of methanogenic communities in the digesters R 4.15 and R 4.16 based on T-RFLP analysis (determined with the restriction enzyme MseI)

It is clear from *Fig. 2* that the composition of the methanogenic communities from the bioreactors R 4.15 and R 4.16 with manure and extruded straw as the used substrates was represented by similar groups as those detected in the reactors R 4.13 and R 4.14. The OLR at three sampling points for the R 4.15 and R 4.16 reactors were 72.1, 68.6, and 69.1 g _oTS_ day ^–1^ , respectively. The members of the genera *Methanosarcina* (70, 54, and 63% of the total abundance in three sampling points, respectively) and *Methanoculleus* (15, 25, and 25% of the total abundance in three sampling points, respectively) were the predominant taxons in the digester R 4.15. Reactor R 4.16, as well as R4.15, was dominated bymembers of thegenera * Methanosarcina* (81, 69, and 51%) and *Methanoculleus* (6, 17, and 28%). Similar to that in the reactors R 4.13 and R 4.14, high abundance of the T-RF peak corresponding to 336 bp was detected; however, the taxonomic group of archaea corresponding to this restriction length profile was not determined. 

These findings substantiate the possibility of effective co-digestion of manure and maize straw, yielding biogas. It has been demonstrated that members of the genera *Methanosarcina* and *Methanoculleus* prevail throughout the fermentation process. In addition, the methanogenic community dynamics during utilization of organic waste has been investigated for the first time. *Methanoculleus * species utilize hydrogen and carbon dioxide for methanogenesis [2], whereas the members of the genus *Methanosarcina* are likely to decompose acetate yielding methane and carbon dioxide or to utilize hydrogen, carbon dioxide, and methylated compounds yielding methane [24]. In all likelihood, the increased concentration of organic acids in the reactors inhibits representatives of the strictly aceticlastic genus *Methanosaeta* and stimulates thedevelopment of *Methanosarcina* spp. [14, 25].

## Abbreviations

T-RFLP: terminal restriction fragment length polymorphism
OTU: operational taxonomic unit
oTS: organic total solids
OLR: organic loading rate
HRT: hydraulic retention time
VFA: volatile fatty acids
